# Reliable reference genes for gene expression analyses under the hypomagnetic field in a migratory insect

**DOI:** 10.3389/fphys.2022.954228

**Published:** 2022-08-08

**Authors:** Ying Zhang, Luying Zeng, Yongji Wei, Ming Zhang, Weidong Pan, Gregory A. Sword, Fei Yang, Fajun Chen, Guijun Wan

**Affiliations:** ^1^ Department of Entomology, College of Plant Protection, Nanjing Agricultural University, Nanjing, China; ^2^ Key Laboratory of Plant Health & Crop Safety, Nanjing Agricultural University, Nanjing, China; ^3^ Beijing Key Laboratory of Bioelectromagnetics, Institute of Electrical Engineering, Chinese Academy of Sciences, Beijing, China; ^4^ Department of Entomology, Texas A&M University, College Station, TX, United States

**Keywords:** migratory insect, magnetic effects, reference gene, gene expression analysis, hypomagnetic field, magnetobiology, magnetoreception, rice planthopper

## Abstract

Manipulating the hypomagnetic field (HMF), which is the absence or significant weakening (<5 μT) of the geomagnetic field (GMF), offers a unique tool to investigate magnetic field effects on organismal physiology, development, behavior and life history. Reverse transcription quantitative polymerase chain reaction (RT-qPCR) has been utilized to study changes in gene expression associated with exposure to the HMF. However, selecting appropriate reference genes (RGs) with confirmed stable expression across environments for RT-qPCR is often underappreciated. Using three algorithms (BestKeeper, NormFinder, and GeNorm), we investigated the expression stability of eight candidate RGs when exposed to the HMF condition versus local GMF during developmental from juveniles to adults in the migratory insect pest, the brown planthopper *Nilaparvata lugens*. During the nymphal stage, *RPL5* & *α-TUB1, EF1-α* & *ARF1*, *RPL5* & *AK*, *EF1-α* & *RPL5*, and *ARF1* & *AK* were suggested as the most stable RG sets in the 1st to 5th instars, respectively. For 1- to 3-day-old adults, *AK* & *ARF1*, *AK* & *α-TUB1, AK* & *ARF1* and *EF1-α* & *RPL5*, *AK* & *α-TUB1*, *AK & EF1-α* were the optimal RG sets for macropterous and brachypterous females, respectively. *ACT1* & *RPL5, RPL5* & *EF1-α, α-TUB1* & *ACT1* and *EF1-α* & *RPL5, ARF1* & *ACT1, ACT1* & *ARF1* were the optimal RG sets for macropterous and brachypterous males, respectively. These results will facilitate accurate gene expression analyses under the HMF in *N. lugens*. The verification approach illustrated in this study highlights the importance of identifying reliable RGs for future empirical studies of magnetobiology (including magnetoreception) that involve magnetic field intensity as a factor.

## 1 Introduction

The geomagnetic field (GMF) provides organisms with protection from solar wind and cosmic radiation, making the Earth hospitable. Living organisms on Earth are immersed in and interact with the GMF. Many animals exploit the vector GMF for orientation and navigation, which is achieved by magnetoreception (Lohmann, 2010; Mouritsen, 2018; Kishkinev et al., 2021; Wynn et al., 2022). Three potential mechanisms, including radical-pair-based quantum compass(Hore and Mouritsen, 2016; Wan et al., 2021; Xu et al., 2021), magnetite-based mechanisms(Kirschvink, 2001; Monteil and Lefevre, 2020) and iron-sulfur cluster assembly 1 (IscA1 or MagR)-Cryptochrome (Cry) magnetosensing complex model (Qin et al., 2016), have received the most attention to date in attempting to explain this enigmatic process.

In addition to magnetoreception which normally functions under the typically-experienced physiological GMF that ranges in strength from ∼ 24 to 66 μT (Alken et al., 2021), bioeffects induced by magnetic fields outside this intensity range on organisms have also been extensively explored (Miyakoshi, 2005; Saunders, 2005; Ghodbane et al., 2013; Zhang et al., 2017; Tian and Pan, 2018; Binhi and Rubin, 2022). A magnetic field that is significantly reduced is usually termed a hypomagnetic field (HMF). An HMF can be found naturally on some planets or satellites (such as Venus, Mars, and the Earth’s moon) (Svedhem et al., 2007; Watters et al., 2007; Berguig et al., 2011) and in the interplanetary space of the solar system. Moreover, it can be artificially achieved on Earth by GMF compensation or shielding strategy using a coils system or high-permeability magnetic material (such as mu-metal or permalloy), respectively. The manipulated HMF is frequently used in mimicking magnetic field intensity during deep space flight or celestial exploration (Binhi and Prato, 2017; Zhang Z. et al., 2021), working as the sham or manipulated treatment group for research into bioeffects induced by changes in field intensity, magnetoreception mechanisms (Fedele et al., 2014a; Binhi and Prato, 2017) and paleomagnetic studies (Qin et al., 2020). Bioeffects of HMFs have been a topic of considerable investigation (Wan et al., 2014; Wan et al., 2015; Binhi and Prato, 2017; Tian and Pan, 2018; Wan et al., 2020b; Zhang and Tian, 2020; Zhang Z. et al., 2021; Xue et al., 2021; Binhi and Rubin, 2022), with demonstrated effects on embryogenesis (Fesenko et al., 2010), development (Mo et al., 2012), reproduction (Wan et al., 2014; Wan et al., 2015), cytoskeleton structure (Mo et al., 2016), nervous system dysfunction and related behavioral outputs (Choleris et al., 2002; Zhang et al., 2004; Binhi and Sarimov, 2009), circadian clockwork (Bliss and Heppner, 1976; Fedele et al., 2014a), migratory regulation (Wan et al., 2015; Wan et al., 2016), and reactive oxygen species levels (Sherrard et al., 2018; Zhang B. et al., 2021). However, the specific mechanisms and signaling pathways underlying phenotypic responses to the HMF remain poorly understood (Binhi and Prato, 2017; 2018).

Gene expression analyses have provided insight into the complex regulatory architecture underlying HMF-triggered bioeffects (Xu et al., 2012; Fedele et al., 2014a; Mo et al., 2014; Wan et al., 2014; Wan et al., 2015; Bae et al., 2016; Fu et al., 2016; Mo et al., 2016; Wan et al., 2016; Agliassa et al., 2018; Agliassa and Maffei, 2019; Zhang B. et al., 2021; Yan et al., 2021) and magnetoreception (Yoshii et al., 2009; Gegear et al., 2010; Xu et al., 2012; Fedele et al., 2014a; Fedele et al., 2014b; Mo et al., 2014; Wan et al., 2014; Wan et al., 2015; Bae et al., 2016; Bazalova et al., 2016; Fu et al., 2016; Mo et al., 2016; Wan et al., 2016; Fitak et al., 2017; Agliassa et al., 2018; Gunther et al., 2018; Wang et al., 2018; Agliassa and Maffei, 2019; Wan et al., 2020a; Hochstoeger et al., 2020; Zhang B. et al., 2021; Gao et al., 2021; Wan et al., 2021; Yan et al., 2021). Reverse transcription quantitative polymerase chain reaction (RT-qPCR) is a powerful tool commonly employed to detect mRNA transcription levels (Derveaux et al., 2010). Accurate normalization is a vital prerequisite for biologically-relevant gene expression analysis. Since no single gene can be used as an internal control under all environmental conditions, the expression stability of the intended reference genes (RGs) has to be verified across environments before use in a formal experiment (Andersen et al., 2004). Uncontrolled variation in detected mRNA amounts can arise due to many factors including tissue type, sampling protocol, total RNA extraction and reverse-transcription efficacy, with unreliable RGs leading to poor reproducibility in genetic and gene expression studies of magnetobiology (Ponton et al., 2011). Insects, with relatively short generation times and powerful molecular toolboxes [e.g., fruit fly (Gegear et al., 2008; Yoshii et al., 2009; Gegear et al., 2010; Foley et al., 2011; Fedele et al., 2014a; Fedele et al., 2014b), monarch butterfly (Gegear et al., 2010; Wan et al., 2021), termite (Gao et al., 2021), cockroach (Bazalova et al., 2016), firebug (Netusil et al., 2021), rice planthopper (Wan et al., 2014; Wan et al., 2015; Wan et al., 2016; Wan et al., 2020a; Zhang Y. et al., 2020; Wan et al., 2020b)]*,* provide excellent models for studying the gene regulatory networks mediating biological responses to changes in magnetic field intensity or direction. However, studies assessing the stability of RGs prior to conducting gene expression analyses in insect magnetobiology studies are rare (Liu et al., 2019).

*Eukaryotic elongation factor 1-α* (*EF1-α*), *18S ribosomal RNA* (*18S*), *actin* (*ACT*), *ADP-ribosylation factor* (*ARF*), *ribosomal protein S* (*RPS*), *tubulin* (*TUB*), *arginine kinase* (*AK*) and *ribosomal protein L* (*RPL*) genes are frequently chosen as internal references (Yuan et al., 2014; Wan et al., 2015; Lu et al., 2018; Wan et al., 2020a) in insect gene expression analyses. The brown planthopper, *Nilaparvata lugens*, a notorious rice pest, exhibits a partial seasonal migration strategy (Menz et al., 2019). Adult *N. lugens* exhibit environmentally-determined wing dimorphism consisting of macropterous migrants and brachypterous residents with enhanced fecundity (Cheng et al., 2003; Guerra, 2011). The candidate magnetite crystals (Pan et al., 2016), as well as putative essential genes in animal magnetoreception [i.e., *Cry1*, *Cry2* (Xu et al., 2016), and *IscA1* (Xu et al., 2019)] involved in the development of these alternative migratory phenotypes, have all been explored. In addition, previous studies have shown migration-related magnetoresponses of nymphal and 1- to 3-day-old *N. lugens* to variation in GMF intensity (Wan et al., 2014; Wan et al., 2020a; Zhang Y. et al., 2020; Zhang and Pan, 2021) [from HMF to moderate magnetic field intensity (Zhang et al., 2017)], establishing *N. lugens*, for which a high-quality genome is available, as a promising unconventional model for magnetobiology (including magnetoreception) study. Therefore, in this study we aimed to investigate the expression stability of eight candidate RGs including *EF1-α*, *18S*, *ACT1*, *ARF1*, *RPS15*, *α*-*TUB1*, *AK* and *RPL5,* with three commonly used normalization algorithms [BestKeeper (Pfaffl et al., 2004), NormFinder (Andersen et al., 2004), GeNorm (Vandesompele et al., 2002)]. We assessed the expression of these potential RGs from the nymphal to the adult stage (including specific developmental stage, sex, and wing morph) of *N. lugens* exposed to HMF (versus the local GMF). We provide the first report to our knowledge of a systematic evaluation with follow-up validation of the reliability of RGs for use in gene expression pathway explorations of magnetobiology (including magnetoreception).

## 2 Materials and methods

### 2.1 Insects

*N. lugens* were originally collected from paddy fields (32°01′50″N, 118°52′25″E) at Nanjing, Jiangsu province of China, during their migration season (mid-to-late July), and were housed indoors to establish a lab colony on susceptible Taichung Native 1 rice seedlings under a 14-h light: 10-h dark (LD) cycle at 26°C and 70%–80% relative humidity (all following assays were under the same environmental conditions except for magnetic fields). The colony was maintained under the local geomagnetic field condition before they were allocated to the experimental magnetic field groups.

### 2.2 Magnetic fields and insect exposures

The geomagnetic field (GMF) intensity at Earth’s surface generally ranges from ∼24 to 66 μT according to the thirteenth generation of the International Geomagnetic Reference Field (Alken et al., 2021). In this experiment, two three-axis DC-type Helmholtz coil systems (external diameter: 1200 mm) were used to mimic the local GMF (mean ± SD; 50000 ± 266 nT) at Nanjing city (32°3′42″N, 118°46′40″E) and the hypomagnetic field (HMF) (mean ± SD; 523 ± 29 nT) at approximately the same inclination and declination within the effective homogeneous areas of 300 mm × 300 mm × 300 mm (<2% heterogeneity). A Faraday cage inside each coil was used to shield the experimental insects from potential anthropogenic electromagnetic noise. The magnetic field parameters were measured and adjusted daily with a fluxgate magnetometer (Model 191A, HONOR TOP Magnetoelectric Technology Co., Ltd., Qingdao, China). The two groups were located in the same room as we did before to secure uniform environmental factors except for magnetic fields (Wan et al., 2020a; Wan et al., 2020b).

Following an established rearing protocol, brown planthoppers, *N. lugens*, were exposed to the HMF versus local GMF treatments from mated F0 females to 3-day-old F1 adults (Wan et al., 2016) that were used in the study. The individuals were maintained under corresponding magnetic conditions throughout the experiments and sampling before being quickly killed in liquid nitrogen for total RNA isolation.

### 2.3 Total RNA extraction and cDNA synthesis

Total RNA was isolated from eight biologically independent pools, each containing five heads of nymphs or adults for each group divided by developmental stage, sex, wing morph and magnetic field intensity. With TRIzol^®^ (Invitrogen; Thermo Fisher Scientific, Inc., Waltham, MA, United States), RNA was extracted from these pooled samples. The quality and quantity of isolated RNA samples were individually analyzed using a NanoDrop 2000 (Thermo Fisher Scientific, Inc., Waltham, MA, United States). Before reverse transcription, each total RNA sample was checked again through electrophoresis in 1% agarose gels. cDNA was synthesized from 100 ng of total RNA in a 20 μl reaction using the PrimeScript RT reagent kit supplemented with a gDNA Eraser (Takara Bio Inc., Dalian, China).

### 2.4 Primer design, testing and RT-qPCR

A total of eight candidate *N. lugens* genes, including *EF1-α*, *18S*, *ACT1*, *ARF1*, *RPS15*, *α*-*TUB1*, *AK* and *RPL5*, were selected as candidate reference genes and their nucleotide sequences were obtained from the GenBank. Primers specific to each gene were designed individually using the Oligo 7 software (Molecular Biology Insights, Inc., Cascade, CO, United States). The synthesis of primers was completed by GeneScript Biotechnology Co., Ltd. (China). A standard curve was generated from a 5-fold dilution of cDNA in a RT-qPCR assay. The PCR efficiency (E) and the correlation coefficient (*R* ^*2*^) were calculated using the slope of the standard curve according to the equation E = [10^−1∕slope^−1] × 100%. Primer specificity was confirmed using melting-curve analysis after RT-qPCR and gel electrophoresis analysis (1.5%) of the amplicon. Primers and amplicon characteristics of the eight candidate reference genes are shown in Table 1.

**TABLE 1:** Primers and amplicon characteristics of the eight candidate reference genes.

Gene (Description)	GenBank no.	Sequence (5′ to 3′; F, forward; R, reverse)	Amplicon length (bp)	Efficiency (%)	Correlation coefficient (*R* ^*2*^)
*EF1-α* (*Elongation factor 1-α*)	KP001173.1	F: ATC​AGC​CAT​TCA​ACT​CAC​CTC​C	98	111.14	0.999
		R: AAC​ACG​ACG​ATA​CAT​GCG​ATA​C			
*18S* (*18S ribosomal RNA*)	JF773148.1	F: TGT​CTG​CTT​AAT​TGC​GAT​AAC​GAA​C	116	109.55	0.996
		R: CCT​CAA​ACT​TCC​ATC​GGC​TTG			
*ACT1* (*Actin 1*)	EU179846.1	F: CTT​CTA​AAC​GCC​AAC​CAC​TCC	110	105.69	0.999
		R: TCA​CCC​GAA​ATC​ACT​CAC​GA			
*ARF1* (*ADP-ribosylation factor 1*)	KT984804.1	F: CCG​CCA​TCT​TTT​CCC​GTT​T	160	112.67	0.993
		R: CAA​TAT​TCT​CAT​CTC​TTT​CTT​GCC​AA			
*RPS15* (*Ribosomal protein S15e*)	FJ810193.1	F: CGC​TCG​CTC​TCA​TCA​AGA​AAC	79	114.98	0.986
		R: TGC​GTC​TTC​ACC​ACT​TCC​G			
*α-TUB1* (*α-tubulin 1*)	KU194637.1	F: TGA​CCG​AGT​TCC​AGA​CTA​ACC​T	107	109.30	0.993
		R: AGA​CAA​CTG​CTC​GTG​GTA​GG			
*AK* (*Arginine kinase*)	KU365925.1	F: ACC​TGT​TCG​ACC​CAA​TCA​T	124	106.64	0.994
		R: ACATCACCGAAGTCCCT			
*RPL5* (*Ribosomal protein L5*)	KX379234.1	F: GAC​CAA​TTA​TGC​CTC​AGC​CTA​C	130	110.76	0.997
		R: CAG​AGC​CTC​CAC​ATT​GTA​CTC​C			

RT-qPCR was performed with an Applied Biosystems^®^ QuantStudio™ 5 Flex Real-Time PCR System (Thermo Fisher Scientific, Inc., Waltham, MA, United States) using SYBR Premix Ex Taq (Tli RNaseH Plus; Takara Bio Inc., Dalian, China), and reactions were conducted in a final volume of 20 μl (including 2 μl of a 1/20 dilution of the cDNA template and primers in a final concentration of 200 nM). Amplifications were performed with an initial 30 s step of 95°C followed by 40 denaturation cycles at 95°C for 5 s and primer annealing at 60°C for 34 s. The melting curve was generated ranging from 60 to 95°C (95°C 15 s; 60°C 1 min, 95°C 15 s).

### 2.5 Analyses of gene expression stability

To evaluate the stability of selected candidate reference genes, BestKeeper, NormFinder, and GeNorm were first employed. For BestKeeper, we mainly adopted SD (the cut-off value of 1) and the coefficient of variance (CV) of the mean Ct values for RG stability evaluation (Pfaffl et al., 2004; Yuan et al., 2014; Wu et al., 2021). NormFinder evaluates the stability of RGs based on intra- and inter-group expression variation and ranks the candidates by the stability values (SV) (Andersen et al., 2004). GeNorm uses the average expression stability (M; the cut-off value of 1.5) to determine the stability of candidate RGs (Vandesompele et al., 2002). According to the original publication*s* of the three algorithms and following practices, stable RGs generally have lower M, SV and SD values (Pfaffl et al., 2004; Yuan et al., 2014; Wu et al., 2021). Thus, a comprehensive ranking was further generated based on the results derived from the geometric mean of these three algorithms (Niu et al., 2015). Besides, GeNorm was also used to determine the optimal number of reference genes. The pairwise variations (Vn/n + 1) were calculated between normalization factors NF_n_ and NF_n + 1_. The Vn/n + 1 value below 0.15 indicates that the addition of the n + 1 RG makes no significant contribution to the normalization (Vandesompele et al., 2002).

### 2.6 Validation assay with suggested reference genes

To verify the reliability of the selected reference genes, the relative expression levels of the *Facilitated trehalose transporter Tret1* (*TRET1*), a conserved transporter for trehalose in insects (Kikawada et al., 2007; Kanamori et al., 2010), were analyzed in 2-day-old brachypterous adults normalized to the reference genes evaluated in this work, including the suggested stable RG (s) (individually or in combination) as well as the least stable RG under HMF versus local GMF. The fold change in gene expression was calculated by the 2^−ΔΔCt^ method (Livak and Schmittgen, 2001). The one-way ANOVA was applied to compare the means of the HMF and GMF at α = 0.05. Effect sizes were estimated using partial η^2^ (small effect: partial η^2^ = 0.01; medium effect: partial η^2^ = 0.06; large effect: partial η^2^ = 0.14) based on the benchmarks of Cohen (Cohen, 2013).

## 3 Results

### 3.1 Expression profile of the candidate reference genes

All primers of the eight candidate reference genes (RGs) (*EF1-α*, *18S*, *ACT1*, *ARF1*, *RPS15*, *α-TUB1*, *AK,* and *RPL5*) showed good amplification specificity with a single amplicon of the expected size by agarose gel electrophoresis analysis of the RT-qPCR products (Supplementary Figure 1A), and with a specific single peak by melting curve analysis (Supplementary Figure 1B). The RT-qPCR efficiency for all eight candidates ranged from 106.64% (*AK*) to 114.98% (*RPS15*), and the correlation coefficients (*R* ^*2*^) varied from 0.986 (*RPS15*) to 0.999 (*EF1-α* and *ACT1*) (Table 1).

**FIGURE 1:**
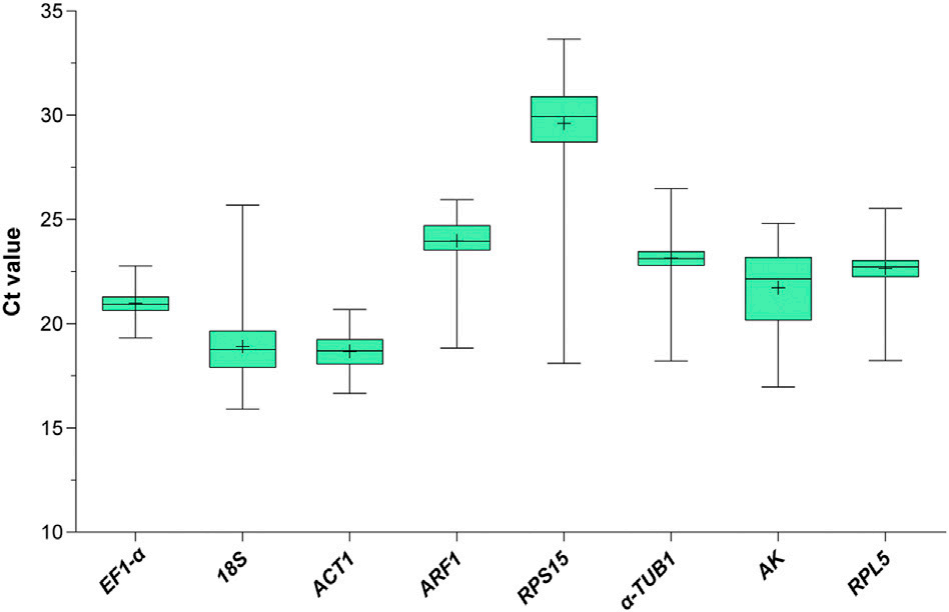
Expression profile of the eight candidate reference genes in all 272 samples under the hypomagnetic field versus local geomagnetic field. The cycle threshold (Ct) distribution is presented as box plots (median, centerline; mean, plus symbol; interquartile range (IQR), box; maximum and minimum values, whiskers).

To assess the stability of the eight candidate RGs across all experimental samples (1st to 5th instar nymphs and 1- to 3-day-old female and male adults with different wing morphs under the HMF versus local GMF), their transcript abundances were determined by the mean threshold cycle (Ct) values through RT-qPCR. As shown in Figure 1, the Ct values of the eight reference genes range from 15.90 to 33.65 (mean Ct, 18.69–29.61). Among them, *RPS15* showed the poorest expression, with the highest mean Ct (29.61) and standard deviation (SD, 2.16) values. *ACT* was the most abundantly expressed gene, with the lowest average Ct and the second-lowest SD (16.85 ± 0.78). *EF1-α* had the lowest SD value (0.66) with a modest mean Ct of 20.98. Moreover, the variability ranking of all genes based on the SD was as follows: *EF1-α* < *ACT1* < *RPL5* < *α-TUB1* < *ARF* < *18S* < *AK* < *RPS15* (Supplementary Table 1), indicating that the transcript expressions of the candidate RGs varied considerably across experimental samples. Thus, it would be essential to select the most reliable RGs for organisms with specific traits (including specific developmental stage, sex and wing morph) under the HMF versus local GMF to ensure accurate gene expression analysis.

### 3.2 Determination of the optimal number of reference genes required for RT-qPCR normalization

**FIGURE 2:**
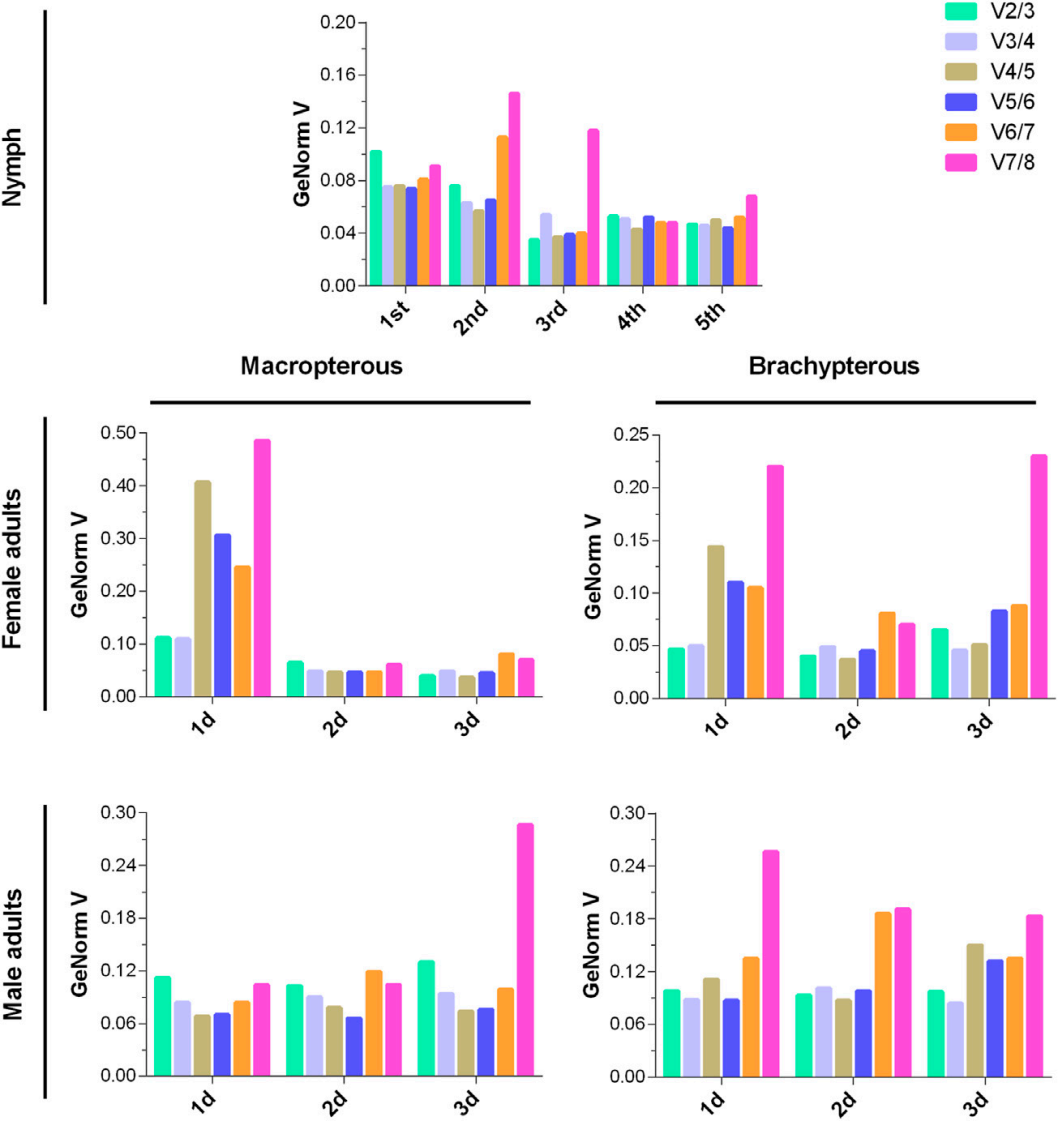
The optimal number of reference genes required for accurate normalization of gene expression of *N. lugens* under the hypomagnetic field versus local geomagnetic field by GeNorm. The pairwise variations (Vn/n + 1) were calculated between normalization factors NF_n_ and NF_n + 1_. The Vn/n + 1 value below 0.15 indicates that the addition of the n + 1 reference gene makes no significant contribution to the normalization.

Before the ranking evaluation, GeNorm was used to determine the optimal number of candidate RGs according to the pairwise variation (Vn/n+1, the cut-off value of 0.15) of normalization factors (Vandesompele et al., 2002). The pairwise variation value below 0.15 indicates that the addition of the n+1 RG makes no significant contribution to the normalization. As depicted in Figure 2, two reference genes (V2/3 < 0.15) were sufficient for accurate normalization of gene expression in the specific developmental stage (1st to 5th instar, 1- to 3-day-old adult), sex (female and male adult), and wing morph (macropterous and brachypterous adults) of *N. lugens* exposed to the HMF condition (versus local GMF).

### 3.3 Expression stability of candidate reference genes in *N. lugens* with specific traits under the hypomagnetic field versus local geomagnetic field

The expression levels of the eight candidate RGs of *N. lugens* with specific traits (including developmental stage, sex and wing morph) exposed to the HMF condition (versus local GMF) were first determined by RT-qPCR and then the BestKeeper, NormFinder, GeNorm as well as comprehensive ranking algorithms were applied to seek the optimal RG(s) in each experimental group. According to the results in Section 2.2, the first two most stable RGs were regarded as the reliable RG combination for accurate gene expression analyses in the corresponding experimental group.

#### 3.3.1 Expression stability of candidate reference genes in *N. lugens* across the nymphal stage

**FIGURE 3:**
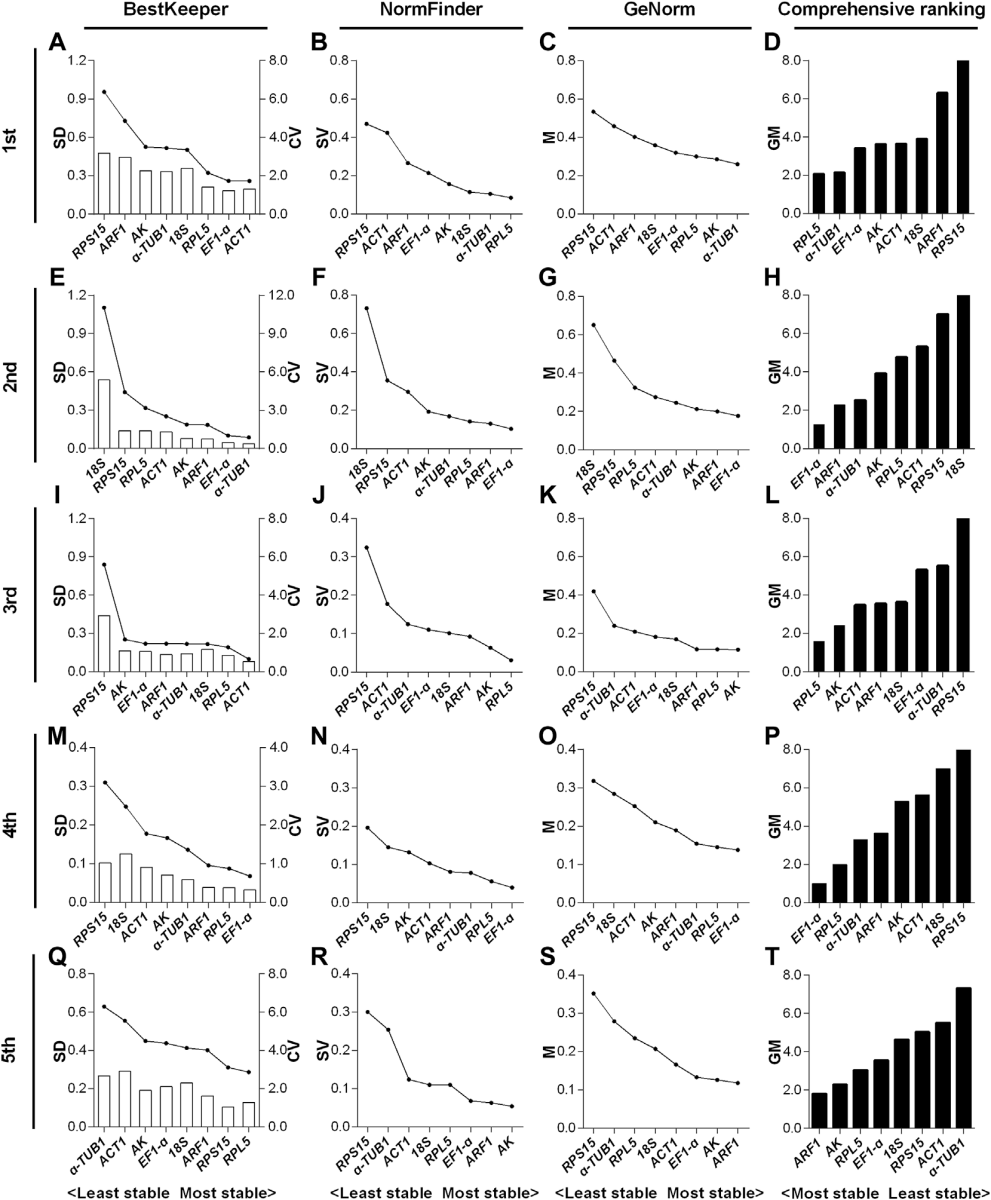
Expression stability evaluation of candidate reference genes respectively for 1st to 5th instar nymphs of *N. lugens* by BestKeeper, NormFinder, GeNorm and comprehensive analyses. Each row of the panel indicates that the experimental samples are respectively from 1st **(A–D)**, 2^nd^ **(E–H)**, 3rd **(I**–**L)**, 4th **(M**–**P)** and 5th **(Q**–**T)** instar nymphs under the hypomagnetic field versus local geomagnetic field. The standard deviation (SD) and coefficient of variation (CV) were given by BestKeeper **(A**,**E**,**I**,**M**,**Q)**. The stability value (SV) was given by NormFinder **(B**,**F**,**J**,**N**,**R)**. The average expression stability (M) was given by GeNorm **(C**,**G**,**K**,**O**,**S)**. The comprehensive ranking was further generated based on the results derived from the geometric mean (GM) of these three algorithms **(D**,**H**,**L**,**P**,**T)**. Stable reference genes generally have lower SD, SV, M and GM values.

For the 1st to 5th instar nymphs, the top two stable RGs under the HMF treatment (versus local GMF) were respectively *RPL5* & *α-TUB1* (Figure 3D), *EF1-α* & *ARF1* (Figure 3H), *RPL5* & *AK* (Figure 3L), *EF1-α* & *RPL5* (Figure 3P) and *ARF1* & *AK* (Figure 3T), while *RPS15* & *ARF1* (Figure 3D), *18S* & *RPS15* (Figure 3H), *RPS15* & *α-TUB1* (Figure 3L), *RPS15* & *18S* (Figure 3P) and *α-TUB1* & *ACT1* (Figure 3T) were, respectively, the two least stable RGs based on BestKeeper, NormFinder, and GeNorm algorithms.

In the 1st instar nymphs, expression stability of *RPL5* was ranked top three by all the three algorithms, while *α-TUB1* ranked 5th by SD value derived from BestKeeper (Figures 3A–C). *EF1-α* & *ARF1* all ranked top three by the three algorithms in the 2nd instar nymphs (Figures 3E–G). *RPL5* ranked top two by all the three algorithms, however, *AK* only ranked the second least stable based on the SD value of BestKeeper in the 3rd instar nymphs (Figures 3I–K). The three algorithms all rated *EF1-α* & *RPL5* as the two most reliable RGs in the 4th instar nymphs (Figures 3M–O). However, in the 5th instar nymphs, inconsistent with the other two algorithms, *AK* only ranked as the 6^th^ most stable RG according to the SD value by BestKeeper (Figures 3Q–S).

#### 3.3.2 Expression stability of candidate reference genes in macropterous and brachypterous female adults

**FIGURE 4:**
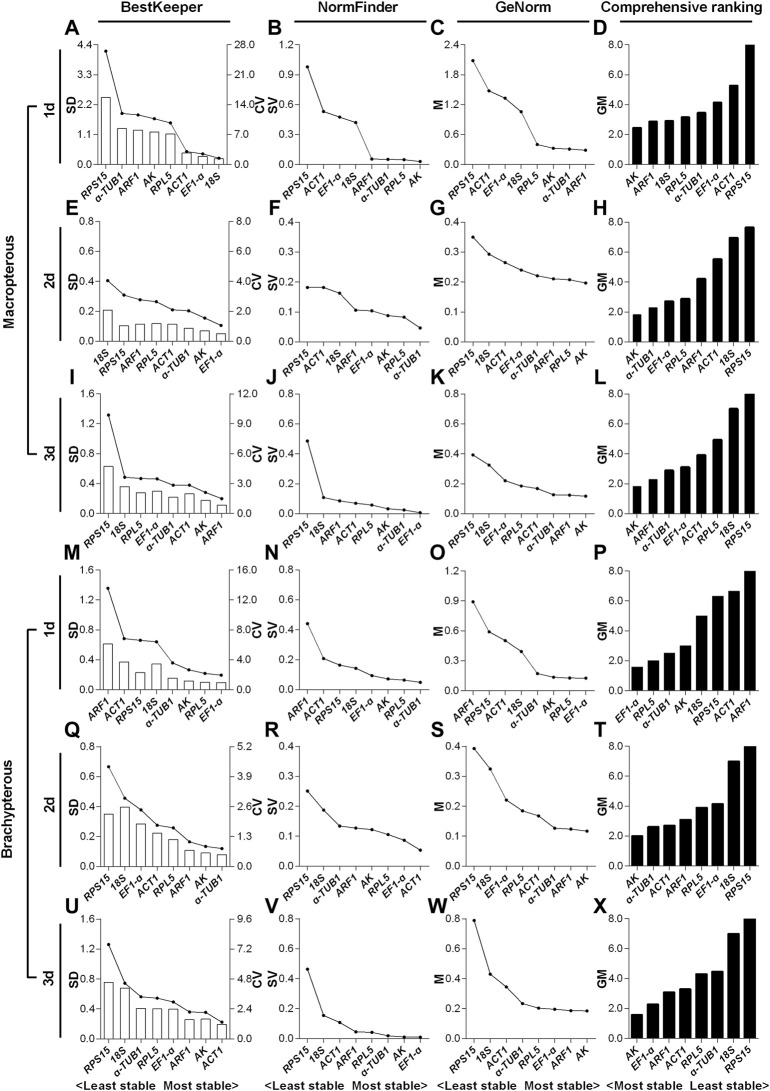
Expression stability evaluation of candidate reference genes respectively for 1- to 3-day-old macropterous and brachypterous female adults of *N. lugens* by BestKeeper, NormFinder, GeNorm and comprehensive analyses. Each row of the panel indicates that the experimental samples are from 1-day-old (1d), 2-day-old (2d) and 3-day-old (3d) macropterous [1d, **(A–D)**; 2d, **(E–H)**; 3d, **(I–L)**] and brachypterous [1d, **(M–P)**; 2d, **(Q–T)**; 3d, **(U–X)**] female adults under the hypomagnetic field versus local geomagnetic field. The standard deviation (SD) and coefficient of variation (CV) were given by BestKeeper **(A,E,I,M,Q,U)**. The stability value (SV) was given by NormFinder **(B,F,J,N,R,V)**. The average expression stability (M) was given by GeNorm **(C,G,K,O,S,W)**. The comprehensive ranking was further generated based on the results derived from the geometric mean (GM) of these three algorithms **(D,H,L,P,T,X)**. Stable reference genes generally have lower SD, SV, M and GM values.

For the 1- to 3-day-old macropterous female adults, the top two stable RG combinations under the HMF treatment (versus local GMF) were respectively *AK* & *ARF1* (Figure 4D), *AK* & *α-TUB1* (Figure 4H) and *AK* & *ARF1* (Figure 4L), while *RPS15* & *ACT1* (Figure 4D), *RPS15* & *18S* (Figure 4H) and *RPS15* & *18S* (Figure 4L) were respectively the two least stable RGs across the same time period evaluated by comprehensive analyses based on BestKeeper, NormFinder, and GeNorm algorithms. Moreover, for the 1- to 3-day-old brachypterous female adults, the top two stable RGs under the HMF treatment (versus local GMF) were respectively *EF1-α* & *RPL5* (Figure 4P), *AK* & *α-TUB1* (Figure 4T) and *AK* & *EF1-α* (Figure 4X), while *ARF1* & *ACT1* (Figure 4P), *RPS15* & *18S* (Figure 4T) and *RPS15* & *18S* (Figure 4X) were respectively the two least stable RGs evaluated across the same time period by comprehensive analyses.

For the macropterous females, *AK* & *ARF1* ranked top 50% out of the eight candidates according to NormFinder and GeNorm algorithms, but were ranked poorly based on SD value of BestKeeper in 1-day-old adults (Figures 4A–C). *AK* & *α-TUB1* all ranked top 50% out of the eight candidates by the three algorithms in 2-day-old adults (Figures 4E–G). In the 3-day-old adults, *AK* & *ARF1* ranked top 50% in the stability evaluation by both BestKeeper and GeNorm, while *ARF1* ranked sixth out of eight based on NormFinder (Figures 4I–K). For the brachypterous females, *EF1-α* & *RPL5* and *AK* & *EF1-α* all ranked top 50% in the expression stability evaluation by all the three algorithms in 1- (Figures 4M–O).and 3-day-old adults (Figures 4U–W). However, in 2-day-old adults, inconsistent with the other two software algorithms, *α-TUB1* only ranked sixth out of eight candidate RGs by NormFinder (Figures 4Q–S).

#### 3.3.3 Expression stability of candidate reference genes in macropterous and brachypterous male adults

**FIGURE 5:**
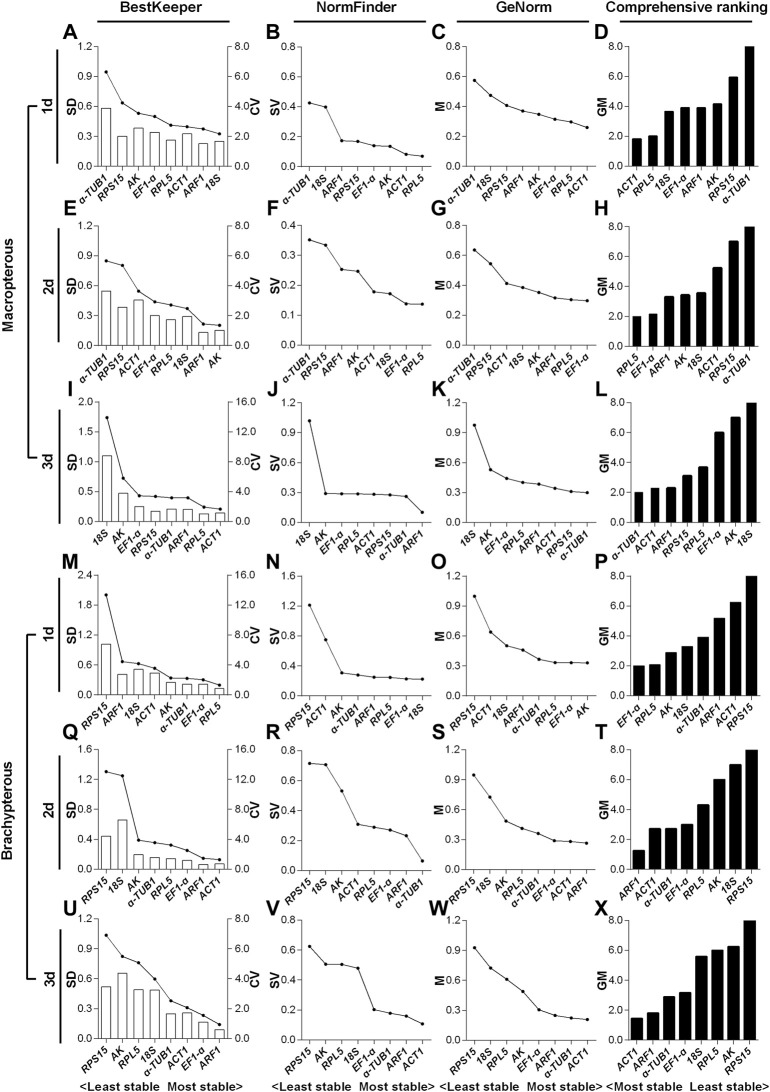
Expression stability evaluation of candidate reference genes respectively for 1- to 3-day-old macropterous and brachypterous male adults of *N. lugens* by BestKeeper, NormFinder, GeNorm and comprehensive analyses. Each row of the panel indicates that the experimental samples are from 1-day-old (1d), 2-day-old (2d) and 3-day-old (3d) macropterous [1d, **(A–D)**; 2d, **(E–H)**; 3d, **(I–L)**] and brachypterous [1d, **(M–P)**; 2d, **(Q–T)**; 3d, **(U–X)**] male adults under the hypomagnetic field versus local geomagnetic field. The standard deviation (SD) and coefficient of variation (CV) were given by BestKeeper **(A,E,I,M,Q,U)**. The stability value (SV) was given by NormFinder **(B,F,J,N,R,V)**. The average expression stability (M) was given by GeNorm **(C,G,K,O,S,W)**. The comprehensive ranking was further generated based on the results derived from the geometric mean (GM) of these three algorithms **(D,H,L,P,T,X)**. Stable reference genes generally have lower SD, SV, M and GM values.

For the 1- to 3-day-old macropterous male adults, the top two stable RGs under the HMF treatment (versus local GMF) were respectively *ACT1* & *RPL5* (Figure 5D), *RPL5* & *EF1-α* (Figure 5H) and *α-TUB1* & *ACT1* (Figure 5L), while across the same time period *α-TUB1* & *RPS15* (Figure 5D), *α-TUB1* & *RPS15* (Figure 5H) and *18S* & *AK* (Figure 5L) were the two least stable RGs evaluated by comprehensive analyses based on BestKeeper, NormFinder, and GeNorm algorithms. Moreover, for the 1- to 3-day-old brachypterous male adults, the top two stable RGs under the HMF treatment (versus local GMF) were respectively *EF1-α* & *RPL5* (Figure 5P), *ARF1* & *ACT1* (Figure 5T) and *ACT1* & *ARF1* (Figure 5X), while across the same time period *RPS15* & *ACT1* (Figure 5P), *RPS15* & *18S* (Figure 5T) and *RPS15* & *AK* (Figure 5X) were the two least stable RGs evaluated by comprehensive analyses.

For the macropterous males, *ACT1* & *RPL5* and *α-TUB1* & *ACT1* respectively ranked top 50% out of the eight candidates in 1- (Figures 5A–C) and 3-day-old (Figures 5I–L) adults according to all three algorithms. *RPL5* & *EF1-α* ranked top two out of the eight candidates by NormFinder and GeNorm, while *EF1-α* ranked only fifth out of the eight based on the SD value of BestKeeper in 2-day-old adults (Figures 5E–G). In addition, for the brachypterous males, *EF1-α* & *RPL5* and *ACT1* & *ARF1* ranked top three out of the eight candidates in 1- (Figures 5M–O) and 3-day-old (Figures 5U–W) adults respectively according to all the three algorithms. *ARF1* & *ACT1* were rated as the top two out of eight stable RGs by both BestKeeper and GeNorm, however, *ACT1* ranked only fifth out of the eight by NormFinder in 2-day-old adults (Figures 5Q–S).

### 3.4 Validation of the selected reliable reference genes

**FIGURE 6:**
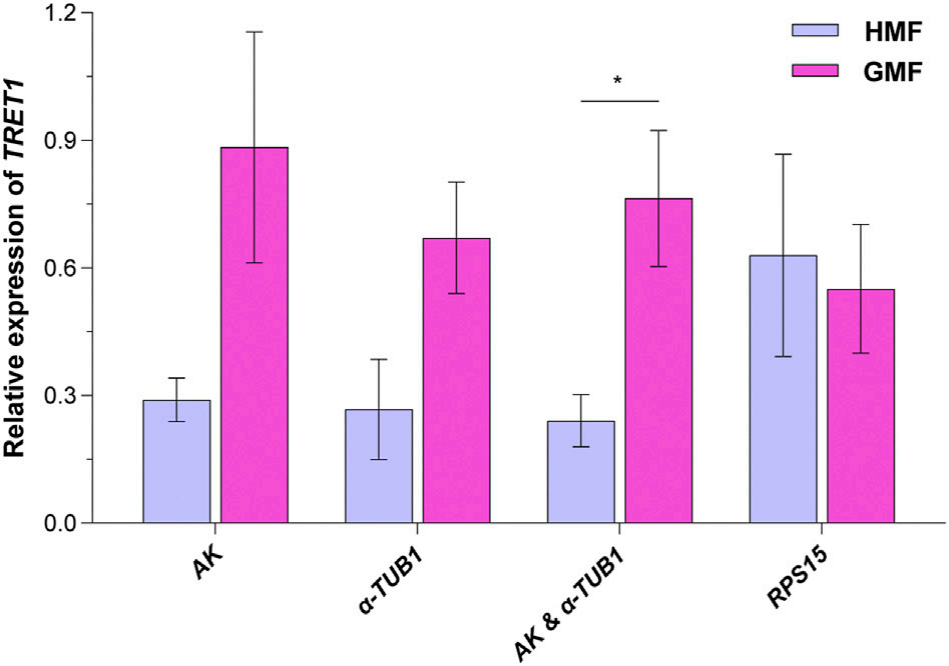
The transcript expression analyses for *TRET1* in 2-day-old brachypterous females normalized to the reference genes evaluated in this work. The top two stable candidate reference genes *AK* and *α-TUB1* (individually or in combination use) and the least stable candidate reference gene *RPS15* were picked for the validation assay under the hypomagnetic field (HMF) versus local geomagnetic field (GMF). Samples of four biologically independent pools were used. The columns represent averages with vertical bars indicating standard errors. Asterisk (*) denotes significant differences between the HMF versus local GMF by one-way ANOVA at *p* < 0.05.

*Facilitated trehalose transporter Tret1* (*TRET1*), a conserved transporter for trehalose in insects (Kikawada et al., 2007; Kanamori et al., 2010), was significantly differentially expressed based on our pilot transcriptome analysis for 2-day-old brachypterous female *N. lugens* subjected to the HMF versus local GMF conditions. We first verified the specificity and performance of the RT-qPCR primer of *TRET1*, as shown in Supplementary Figure 2. Then, to validate the selected reference genes, the relative expression levels of the target gene *TRET1* in 2-day-old brachypterous females normalized to the reference genes evaluated in this work, including the top two stable RGs *AK* and *α-TUB1* (individually or in combination use) and the least stable *RPS15*, were assessed under the HMF versus local GMF using RT-qPCR. Consistently, 2-day-old brachypterous females showed significant differences in *TRET1* transcript expression levels between the HMF and local GMF groups using the suggested *AK* and *α-TUB1* as RGs (*F*_1, 6_ = 9.376; *p* = 0.022; partial *η* ^2^ = 0.61). When using *AK* (*F*_1, 6_ = 4.629; *p* = 0.075; partial *η* ^2^ = 0.44) or *α-TUB1* (*F*_1, 6_ = 5.247; *p* = 0.062; partial *η* ^2^ = 0.47) as RG individually, consistent *TRET1* transcript expression patterns can also be found, although the difference was not significant at the *p* < 0.05 level between the two magnetic field conditions. However, comparable expression levels of *TRET1,* but in a different pattern, were found between groups when the least stable *RPS15* (*F*_1, 6_ = 0.079; *p* = 0.788; partial *η* ^2^ = 0.01) was used as the only RG (Figure 6).

## 4 Discussion

Static magnetic fields are generally classified as hypomagnetic (HMF) (<5 μT) (Zhang B. et al., 2021), weak (<1mT), moderate (1 mT–1 T), strong (1–20 T) and ultra-strong (>20 T) magnetic fields (Zhang et al., 2017). The HMF offers a unique option to help shed light on how magnetic fields (especially the GMF) influence life and the potential mechanisms relevant to the phenotypic effects of magnetic fields as well as magnetoreception mechanisms. Combined with multi-omic and reverse genetic tools, gene expression analysis plays a crucial role in uncovering the complex gene regulatory architecture underlying the HMF-triggered bioeffects and magnetoreception. However, few magnetobiology studies have included a preliminary stability assessment of the reference genes used in gene expression analyses of the target genes under investigation (Di et al., 2011; Fu et al., 2016; Agliassa et al., 2018; Agliassa and Maffei, 2019; Liu et al., 2019; El May et al., 2021). Using the migratory *N. lugens,* which has the potential to be an unconventional insect model for magnetobiology and magnetoreception, we systematically assessed the stability of eight selected candidate reference genes (RGs) across developmental stages, sexes, and wing morphs with three widely used algorithms [BestKeeper (Pfaffl et al., 2004), NormFinder (Andersen et al., 2004), and GeNorm (Vandesompele et al., 2002)]. A follow-up validation assay with 2-day-old brachypterous females targeting *TRET1*, a conserved trehalose transporter, was then conducted to test the reliability of the suggested RG (s) (Kikawada et al., 2007; Kanamori et al., 2010).

According to the GeNorm, two reference genes were suggested here for normalization of gene expression in *N. lugens* under the two magnetic field groups, consistent with that of *Laodelphax striatellus*, another notorious species of rice planthopper (Liu et al., 2019). In the validation assay, the difference in transcript expression of target gene *TRET1,* which functions in mediating the trehalose exchanges among various tissues in insects (Kikawada et al., 2007; Kanamori et al., 2010), reached a significant level between the HMF versus GMF only when using the combination of the suggested top two stable RGs. This result is consistent with our previous work showing that trehalose levels varied significantly between two different magnetic field intensities. Moreover, the validation assay also indicated that combining two stable RGs rather than a single one increased the effect size, further supporting the importance of introducing another stable RG to secure more accurate experimental results. Although there have different screening principles and emphases (Vandesompele et al., 2002; Andersen et al., 2004; Pfaffl et al., 2004) for expression stability analysis amongst BestKeeper (Pfaffl et al., 2004), NormFinder (Andersen et al., 2004), and GeNorm (Vandesompele et al., 2002), the recommended top two most stable RGs were consistent in most conditions. Nevertheless, a comprehensive analysis was still further applied based on the results derived from the geometric mean of these three algorithms, which offered good comprehensive ranking performance based on common practices (Xie et al., 2012; Niu et al., 2015; Zhang Z. et al., 2020) and our validation assay.

Previous studies *in vitro* have shown that changes in GMF intensity [including the strong magnetic field (Qian et al., 2010) and HMF (Wang et al., 2008; Mo et al., 2016)] can affect cytoskeleton and cytoskeleton-associated genes, which may be due to quantum effects (Zadeh-Haghighi and Simon, 2022). In particular, the *in vitro* assembly of TUB (Wang et al., 2008) and F-ACT (Mo et al., 2016) at the protein level can be affected by the HMF. However, our previous work suggested *α2-TUB* as the most stable RG in newly emerged brachypterous male adults of *L. striatellus*. The transcript expression stability of *ACT1* and *α-TUB1* of *N. lugens* was also assessed in the current study. According to the expression variability (SD) of the eight candidate RGs in all 272 samples under the HMF versus GMF, *ACT1* and *α-TUB1* were respectively ranked as the 2nd and 4th most stable RGs (Supplementary Table 1). Although the expression stability of *ACT1* was ranked poorly during the nymphal stage and in 1-3-day-old macropterous female adults for most situations, its expression stability performed well in the majority of the rest groups based on the three algorithms and corresponding comprehensive rankings. For *α-TUB1*, its evaluated expression stability also varied across developmental stages, sexes, and wing morphs (Figures 3–5). Moreover, our validation assay using *α-TUB1* as one of the top two stable RGs in 2-day-old brachypterous females further affirmed the constant expression of *α-TUB1* between the HMF versus GMF as well as the reliability of the expression stability ranking scored by the algorithms (Figure 6). All these results indicate that the reported effects triggered by the HMF on F-ACT and TUB are likely to exert only at the protein level or in a trait-specific way.

As with the current study, the only two other systematic reference gene selection studies, to our knowledge, also found that commonly used housekeeping genes are not always consistently expressed between magnetic field intensities *in vivo* (Liu et al., 2019) and *in vitro* (Di et al., 2011). To some extent, as an extension of our previous RG selection work with brachypterous female and male *L. striatellus* (Liu et al., 2019)*,* the current study with *N. lugens* further showed that the assessed RG expression stability varied across not only sex but also developmental stage and wing morph under the HMF versus local GMF. Having a closer look, the top two stable RGs frequently varied while *RPS15* remained to be ranked as the last three out of eight across different groups. Thus, it should be noted that even though *PRS15* was reported as the most suitable RT-qPCR reference gene for *N. lugens* at different developmental stages (Yuan et al., 2014), it is not a reliable reference gene under the HMF condition. When adopting *PRS15* as the only RG for magnetic field intensity treatment, comparable expression levels of *TRET1* in a contrary pattern were found compared to using suggested stable RG(s). Interestingly, unlike *RPS15*, another ribosomal protein gene widely used as the housekeeping gene, *RPL5*, was scored much better by the algorithms, which may be due to the difference in sensitivity to magnetic field intensity change regarding their ribosome-independent functions (Zhou et al., 2015).

Overall, increasing evidence indicates that the magnetic field intensity is a tricky environmental factor to control for and requires more attention in the design and analysis of gene expression studies (Makinistian and Belyaev, 2018). The gene expression stability assay presented here highlights the potential importance of using reliable RG(s) in gene expression investigations of magnetobioloy including magnetoreception. This study provides a basis for more reliable future studies as we unveil the potential signal pathways underlying responses to changes in magnetic field intensity in the important migratory pest, *N. lugens*.

## Data Availability

The original contributions presented in the study are included in the article/Supplementary Materials, further inquiries can be directed to the corresponding author.
